# Comparison of Hernia Sac Transection and Full Sac Reduction for the Treatment of Inguinal Hernias: A Systematic Review and Meta‐Analysis of Clinical Trials

**DOI:** 10.1002/wjs.12474

**Published:** 2025-01-24

**Authors:** Roberto Cirocchi, Georgi I. Popivanov, Maria Chiara Cianci, Antonino Morabito, Matteo Matteucci, Sara Lauricella, Diletta Cassini, Carlo Boselli, Ivan Szergyuk, Giovanni Domenico Tebala, Antonia Rizzuto, Paolo Bruzzone

**Affiliations:** ^1^ Department of Medicine and General Surgery University of Perugia Perugia Italy; ^2^ Department of Surgery Medical Military Academy of Sofia Sofia Bulgaria; ^3^ University of Florence Meyer Children's Hospital Florence Italy; ^4^ Department of Medicine and General Surgery University of Milan Milan Italy; ^5^ Colorectal Surgery Division Department of Surgery Fondazione IRCCS Istituto Nazionale dei Tumori Milan Italy; ^6^ General and Emergency Surgery Sesto San Giovanni Hospital Milan Italy; ^7^ Faculty of Medicine Jagiellonian University Medical College Krakow Poland; ^8^ Department of Digestive and Emergency Surgery Hospital of Santa Maria of Terni Terni Italy; ^9^ Department of Medical and Surgical Sciences Magna Græcia University Catanzaro Italy; ^10^ Dipartimento di Chirurgia Generale e Specialistica Sapienza University of Rome Rome Italy

**Keywords:** hernia sac reduction, hernia sac transection, inguinal hernia

## Abstract

**Background:**

The history of inguinal hernia repair has been marked by the description of several therapies over ages, each with its own approach to managing the hernial sac. An analysis of hernia sac transection (with or without high ligation) versus reduction (invagination) in adults who underwent Lichtenstein open tension‐free inguinal hernia repair and in adult and pediatric patients who underwent suture repair has been the primary aim of this systematic review and meta‐analysis.

**Methods:**

The authors conducted a comprehensive review and meta‐analysis. A comprehensive literature search yielded 15 publications, consisting of 12 randomized controlled trials (RCTs) including 1598 patients and 3 controlled clinical trials (CCTs) including 243 patients. In total, the included patients amounted to 1.841.

**Results:**

Analysis of the data revealed a lower rate of recurrence in patients who had sac reduction (0.35% in randomized controlled trials and 0 in clinical trials) compared to patients who had sac excision and ligation (0.86% in randomized controlled trials and 0.93% in clinical trials). However, this difference was not statistically significant (RCTs: relative risk 2.94 [0.30, 29.24]—CCTs: relative risk 4.46 [0.18, 111.36]).

**Conclusion:**

The reduction of sacs does not result in a statistically significant decrease in recurrence compared to patients who underwent sac excision and subsequent ligation. This study has demonstrated that the various courses of treatment for the inguinal hernia sac have similar primary and secondary outcomes in both adult and pediatric patients.

## Graphical abstract

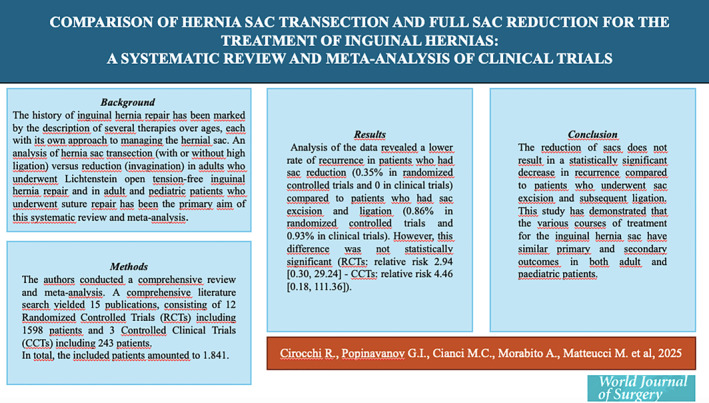

## 1 Introduction

The repair of an open hernia begins with the incision of the inguinal canal and the separation of the hernial sac from the spermatic cord [1, 2, 3]. However, the subsequent course of action remains a subject of debate: many surgeons propose a straightforward removal of the sac (herniotomy), while others favor the invagination of the sac [4]. High sac ligation proponents argue that this method is necessary to decrease the frequency of hernia recurrences. The procedure in question has been associated with avoidable postoperative pain, an increased incidence of postoperative ileus and hemorrhage at peritoneal incisions, and the potential for fibrotic adhesions between a peritoneal scar and the small intestine [5]. The proposal of high ligation and excision of the indirect hernia sac as a crucial measure to decrease recurrences was initially put up by Edoardo Bassini. Today, however, the emphasis has changed from managing the sac to rectifying the abnormality. Laparoscopic hernia repairs have demonstrated a recent breakthrough by eliminating the need for sac ligation and instead inverting or excising the sac, resulting in similar rates of recurrence [6]. The Shouldice procedure involves opening, inspecting, and either excising or inverting the indirect hernial sac without ligation in a no‐mesh pure tissue repair. Optional management strategies for the indirect hernial sac in the Shouldice technique had a lesser impact on recurrence compared to open mesh repairs [4]. Rutkow [7] cautioned against surgically accessing the hernia sac for visual examination. Furthermore, he proposed that by tying the sac, it may cause localized symptoms similar to peritoneal inflammation, as the sac is derived from the very sensitive peritoneum. Therefore, a significant portion of the postoperative discomfort and agony might be ascribed to this artificial peritonitis. Due to the lack of evidence supporting the potential improvement in the recurrence rate with high ligation, Rutkow's mesh plug repair avoids it. Similarly, in the Lichtenstein repair, the sac is inverted or removed instead of being ligated.

The primary aim of this systematic review and meta‐analysis is to examine the differences between hernia sac transection (with or without high ligation) and reduction (invagination) in children and adults who underwent suture repair and Lichtenstein open tension‐free inguinal hernia repair. Our hypothesis is that invagination offers notable benefits in terms of reducing postoperative pain without increasing the occurrence of recurrences, compared to hernia sac transection.

## 2 Methods

The study protocol for this systematic review and meta‐analysis was registered in PROSPERO (http://www.crd.york.ac.uk/prospero) (CRD42022306845). We conducted a systematic review of literature updated on 1 October 2022 according to the Preferred Reporting Items for Systematic Reviews and Meta‐Analyses (PRISMA) guidelines.

### 2.1 Types of Studies

All randomized controlled trials (RCTs) and clinical control studies (CCT) were included, regardless of their publishing status, language of publication, or if outcomes were provided.

### 2.2 Types of Participants

Our study encompassed both children (0–18 years) and adults with inguinal hernia who received any type of suture repair, as well as adults who underwent Lichtenstein open tension‐free repair for primary inguinal hernia. There were no limitations based on race or sex. We excluded studies that combined data from both adults and children and instead included only studies where data were reported separately for each subgroup (adults and children). We have included studies of all types of inguinal hernias (direct or indirect) without considering their size or bilaterality. Irrespective of the length of follow‐up, we included all studies that reported data on the primary or secondary outcomes.

### 2.3 Types of Interventions

Patients were undergone at open inguinal hernia repair and sac transection with or without ligation. Only patients who had undergone elective repairs were included, whereas those who underwent emergency repairs were excluded.

### 2.4 Types of Outcome Measures

#### 2.4.1 Primary Outcomes

- Recurrence of clinical hernia with a minimum follow‐up period of 12 months.
- Groin discomfort following surgery is assessed with the visual analog scale (VAS) at three time points: 24 h, after 1 week, and 6 months or more.

#### 2.4.2 Secondary Outcomes

Adverse effects occurring within 30 days following surgery include hematoma, wound infection, seroma, scrotal edema, urine retention, and testicular atrophy.

### 2.5 Search Methods for Identification of Studies

We conducted searches in the following electronic databases without applying any limitations on the language of publication:

– The Cochrane Central Register of Controlled Trials (CENTRAL) can be accessed through the Cochrane Register of Studies Online (CRSO).
– MEDLINE (ovid MEDLINE ALL from 1946 to the current daily update)
– SCOPUS
– Web of Science (WOS) index

Additional studies were identified by searching the bibliography of selected trials using the PubMed feature “related articles”. Gray literature analysis was conducted by searching on Google Books (https://books.google.com). Furthermore, we conducted a search in the subsequent databases:

– The official website of ClinicalTrials.gov
– www.who.int/trialsearch/.

### 2.6 Data Collection and Analysis

After conducting bibliographic research in the predefined databases, two authors (R.C. and P.B.) independently evaluated the titles and abstracts of the selected studies. These two authors obtained the full‐text of all potentially eligible trials and independently screened these full‐texts for potential inclusion. We do not find multiple publications on the same study population.

### 2.7 Data Extraction and Management

Using a data extraction form developed and pilot‐tested by the authors, two authors (R.C. and M.M.) independently collected data from the included studies. For each study, the following information has been extracted: the surname of the first author and the year of publication, the country of the hospital where the study was conducted, the type of study and the number of patients included, the setting, participant characteristics (including age, type, and size of hernia), study eligibility criteria, details of the interventions (hernia repair with or without mesh), the outcomes assessed, the source of study funding, and any conflicts of interest stated by the investigators. The extracted data have been entered into the Review Manager 5.4 software, and the combined data have been analyzed to synthesize the results.

### 2.8 Measures of Treatment Effect

Categorical outcomes were analyzed using pooled relative risk (RR) with 95% confidence intervals (CIs), whereas continuous outcomes were analyzed using mean differences (MD) or standardized mean differences with 95% confidence intervals (CIs).

### 2.9 Assessment of Heterogeneity

Statistical heterogeneity was assessed by employing both the I^2^ statistic and the ϳ^2^ test. In the ϳ^2^ test, we determined that heterogeneity was present when *p*‐values were less than 0.10. Our interpretation of I^2^ was based on the guidelines provided in the Cochrane Handbook for Systematic Reviews of Interventions. Specifically, we considered 0%–40% to be insignificant, 30%–60% to indicate moderate heterogeneity, 50%–90% to indicate significant heterogeneity, and 75%–100% to indicate significant heterogeneity.

### 2.10 Data Synthesis

We conducted statistical analysis using the Review Manager 5.4.1 software (Review Manager 2020). We employed random‐effects models to calculate effect sizes. We displayed all findings together with their corresponding 95% confidence intervals (CIs). We visually represented the results of meta‐analyses for each outcome as forest plots.

## 3 Results of Systematic Review

**FIGURE 1:**
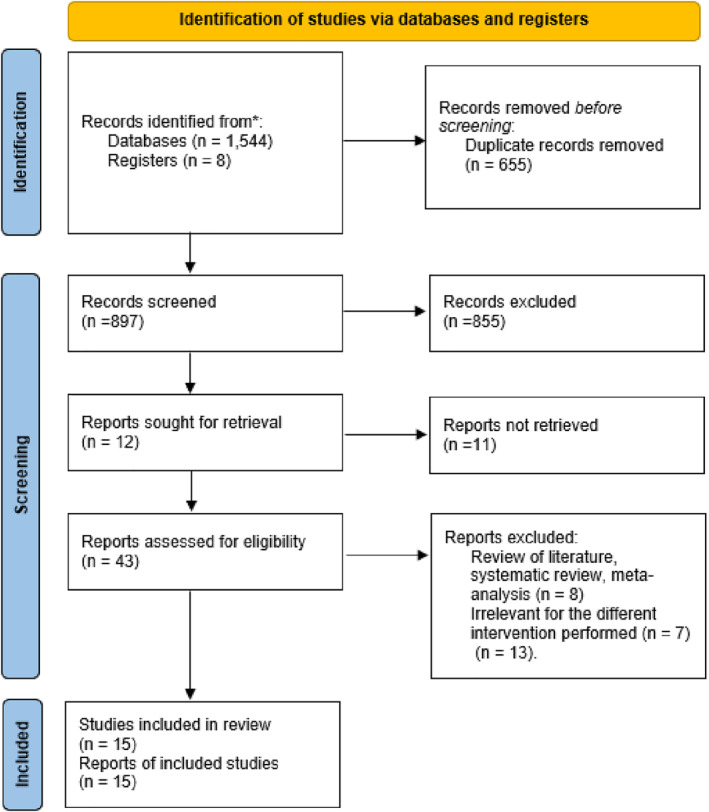
PRISMA flow chart for systematic review and meta‐analysis.

The PRISMA flow chart for the systematic review was reported in Figure 1.

The initial search produced 1552 potentially relevant articles. After the exclusion of duplicates, the titles and abstracts were screened for relevance, an evaluation of 43 full text was performed, and successively 28 studies were excluded and 15 publications [8, 9, 10, 11, 12, 13, 14, 15, 16, 17, 18, 19, 20, 21, 22] suitable for inclusion in this meta‐analysis: 12 randomized controlled trials (1598 patients) and 3 CCTs (243 patients) (Table 1).

**TABLE 1:** Characteristics of the included studies.

Study, year, country	Journal name	Nation	Study design	Length of follow‐up	Included patients	Type of repair		Patients enrolled			Age	Male sex
											Mean (SD)	number/total (%)
						Technique	Sac excision	Total	Sac Invagination	Sac excision and ligation	(Invagination vs. ligation)	
Salimi et al, 2023	Iranian Journal of Pediatric Surgery	Iran	CCT	5 years	Pediatric patients with sliding hernia	Hernia repair without mesh	with ligation	153 patients (153 hernias)	91 patients (91 hernias)	62 patients (62 hernias)	315.2 ± 280.04 (days)	233.16 ± 193.87 (days)
Jain et al, 2023	International Journal of Pharmaceutical and Clinical Research	India	RCT	3 months	Adult patients with indirect inguinal hernia	Lichtenstein repair	With ligation	60 patients (60 hernias)	30 patients (30 hernias)	30 patients (30 hernias)	44.7 ± 3.8 versus 48.4 ± 5.4	30/30 (100%) versus 29/30 (9.6%)
Majithiya et al, 2022	International Journal of Scientific Research	India	CCT	1 year	Adult patients with indirect inguinal hernia	Lichtenstein repair	With ligation	38 patients (38 hernias)	18 patients (18 hernias)	20 patients (20 hernias)	NR	NR
Haider et al, 2022	International Journal of Current Advanced Research	India	RCT	48 h	Adult patients with indirect or direct inguinal hernia	Lichtenstein repair	With ligation	70 patients (70 hernias)	35 patients (35 hernias)	35 patients (35 hernias)	38.81 (11.71)	NR
Ciftci et al, 2022	Hernia	Turkey	RCT	18 months	Adult patients with indirect inguinal hernia	Lichtenstein repair	With ligation	93 patients (93 hernias)	49 patients (49 hernias)	44 patients (44 hernias)	56.02 ± 13.43 versus 58.31 ± 13.17	44/49 (90%) versus 43/44 (97%)
Ahamed et al, 2021	International Journal of Current Advanced Research	India	RCT	3 years	Adult patients with indirect or direct inguinal hernia	Lichtenstein repair	With ligation	200 patients (200 hernias)	100 patients (100 hernias)	100 patients (100 hernias)	44.8 ± 8.9 versus 46 ± 8.8	NR
Dave et al, 2021	International Journal of Science and Research	India	RCT	6 months	Adult patients with indirect inguinal hernia	Lichtenstein repair	With ligation	50 patients (50 hernias)	25 patients (25 hernias)	25 patients (25 hernias)	NR	NR
Gupta et al, 2019	International Surgery Journal	India	RCT	6 months	Adult patients with indirect inguinal hernia	Lichtenstein repair	With ligation	50 patients (50 hernias)	25 patients (25 hernias)	25 patients (25 hernias)	46.76 ± 16.13 versus 44.97 ± 14.34	NR
Sharma et al, 2019	Annals of Royal College of Surgeons	India	RCT	12 months	Adult patients with indirect inguinal hernia	Lichtenstein repair	With ligation	70 patients (70 hernias)	35 patients (35 hernias)	35 patients (35 hernias)	37.71 ± 15.03 versus 37.63 ± 13.55	35/35 (100%) versus 35/35 (100%)
Ranga et al. 2016	Journal of Evolution of Medical and Dental Sciences	India	CCT	2 years	Adult patients with indirect inguinal hernia	Lichtenstein repair	With ligation	50 patients (50 hernias)	25 patients (25 hernias)	25 patients (25 hernias)	43.96 ± 17.019 versus 35.8 ± 17.102	NR
Rafiei et al. 2015	Advanced Biomedical Researcher	Iran	RCT	3 months	Pediatric patients	Hernia repair without mesh	With ligation	104 patients (104 hernias)	52 patients (52 hernias)	52 patients (52 hernias)	1 month ‐ 8 years	93/104 (89%)
Othamn et al. 2014	Hernia	Egypt	RCT	78 months	Adult patients with indirect inguinal hernia	Lichtenstein repair	With ligation	103 patients (111 hernias)	50 patients (54 hernias)	53 patients (57 hernias)	48.40 ± 21.01 versus 48.36 ± 19.90	50/50 (100%) versus 52/53 (98%)
Zaman et al. 2013	Journal of Fatima Jinnah Medical University	Pakistan	RCT	NR	Pediatric patients	Hernia repair without mesh	With ligation	100 patients (100 hernias)	50 patients (50 hernias)	50 patients (50 hernias)	40 ± 12.07	99/100 (99%)
Tabrizian et al. 2013	Journal of Pediatric Surgery	Iran	RCT	1–1.5 years	Pediatric patients	Hernia repair without mesh	With ligation	100 patients (121 hernias)	30 hernias	91 hernias	NR	87/100 (87%)
Delikoukos et al. 2007	Hernia	Greece	RCT	1 month	Adult patients with indirect inguinal hernia	Lichtenstein repair	With ligation	477 patients (477 hernias)	239 patients (239 hernias)	238 patients (238 hernias)	61.2 ± 9.8 versus 60.5 ± 10.6	211/239 (88%) versus 209/238 (88%)

Abbreviations: CCT, Clinical controlled trial; E + HL, excision with high ligature of the hernial sac; HL ‐ E, excision without ligature of hernia sac; NR, not reported; SD, standard deviation; RCT, Randomized controlled trial; RS, reduction of the hernial sac.

A total of 1841 patients were involved in 15 investigations. Among them, 935 patients had excision of the hernial sac with high ligature, whereas 906 patients had invagination of the hernial sac. The majority of the patients included were adults (1.384 patients), while just a small number were adolescents (457 patients).

Each study, with the exception of Zaman [22], provided data on the duration of follow‐up. Some studies included a follow‐up check in the immediate postoperative days, namely between 1 and 10 days after surgery, whereas in other cases, the follow‐up time extended to weeks, months, or even years after surgery. Consequently, this variable could not be compared across the studies.

### 3.1 Peculiarities of Study Patients

All 15 [8, 9, 10, 11, 12, 13, 14, 15, 16, 17, 18, 19, 20, 21, 22] included studies provided some information concerning patients' ages, such as mean age or interval of ages; all studies excepts Gupta [12] provided data concerning patients' sex: 46.411 males and 3496 women. Only Delikoukos [11] and Sharma [20] reported BMI as a mean value. Only Rafiei [17] evaluated surgical risk using the American Society of Anesthesiologists ASA physical status classification system. Only 4 papers out of 13 reported patients' levels of comorbidities (Delikoukos [11] specified the kind of comorbidity—Othman [16] e Ciftci [9] reported only the number of patients with comorbidities. Zaman [22] only quoted that diabetic patients were excluded from his study).

### 3.2 Assessment of Risk of Bias in Included Studies

**FIGURE 2:**
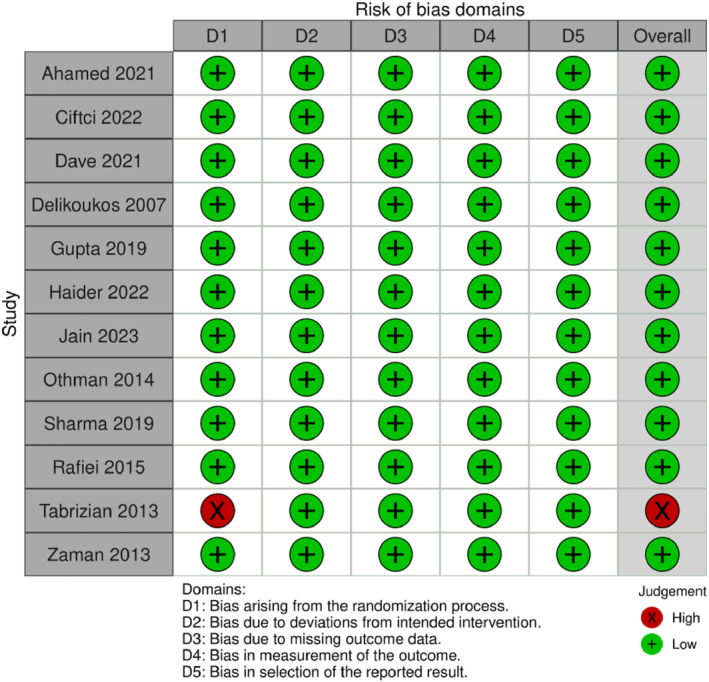
Risk of bias domains for included studies RCTs using the Rob2 tool.

**FIGURE 3:**
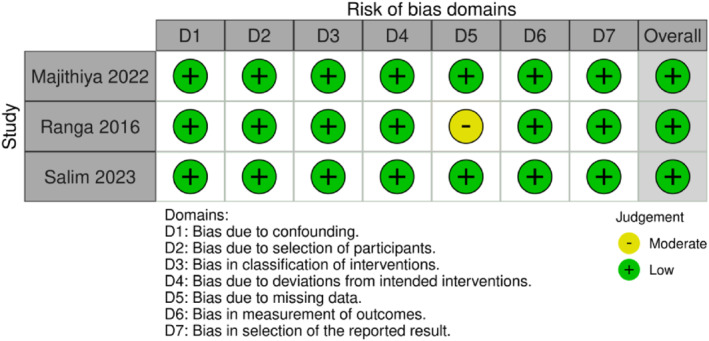
Risk of bias domains for included studies CCTs using the Rob1.

Each study chosen for inclusion underwent separate assessments for bias by two authors (R.C. and G.P.). To evaluate the risk of bias in randomized controlled trials (RCTs), we used version two of the Cochrane ‘risk of bias' tool (RoB 2), as outlined in Chapter 8 of the Cochrane Handbook for Systematic Reviews of Interventions (Figure 2). For the assessment of the risk of bias in CCTs, we used the ‘risk of bias' tool (RoB 1) (Figure 3).

## 4 Data Analysis

### 4.1 Primary Outcomes

#### 4.1.1 Postoperative Recurrence at 1 Year or Longer After the Initial Hernia Surgery

**FIGURE 4:**
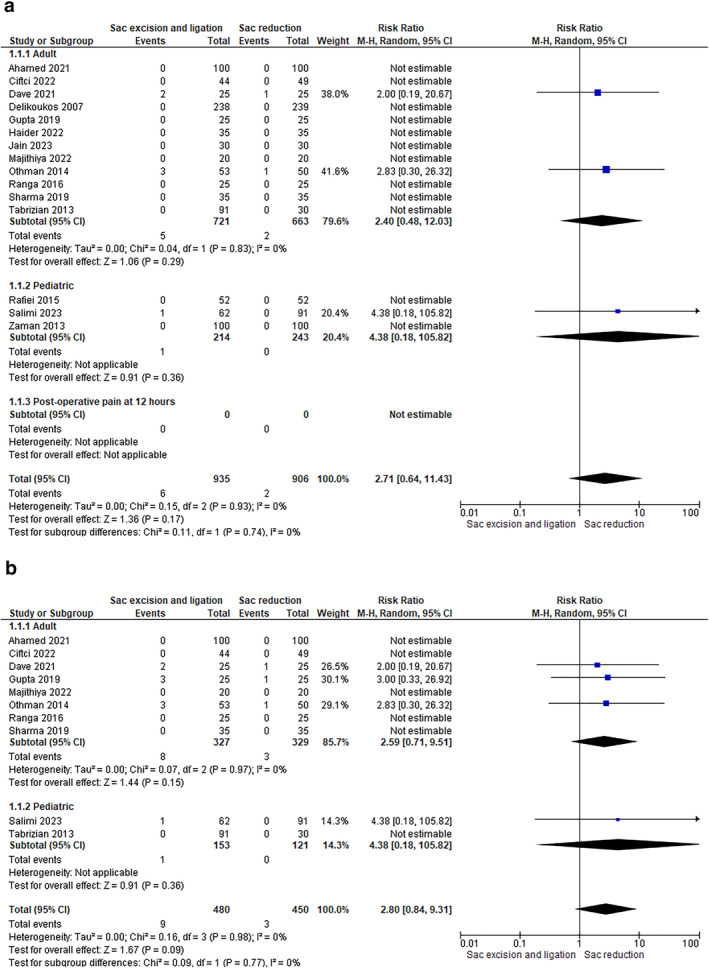
(a) Postoperative recurrence at 1 year or longer after the initial hernia surgery. (b) Postoperative recurrence at 1 year or longer in different groups of age (adult vs. pediatric).

The reported outcome has been examined in all studies included in this systematic review and meta‐analysis (15 studies–1841 patients). The data analysis of both RCTs and CCTs has shown a reduction in recurrence in patients who had only sac reduction (0.26% in RCTs and 0 in CCTs) compared to patients who underwent sac excision and ligation (0.95% in RCTs and 0.93% in CCTs). However, this difference was not statistically significant (RCTs: RR 2.40 [0.48, 12.03]–CCTs: RR 4.38 [0.18, 105.82]) (Figure 4a).

The subgroup analysis based on the age of patients yielded similar results: Older patients who underwent sac reduction had fewer recurrences (0.91% in adult patients and 0 in pediatric patients) compared to those who underwent sac excision and ligation (2.45% in adult patients and 0.65% in pediatric patients). However, this difference was not statistically significant (adults: RR 2.59 [0.71, 9.51]–pediatrics: RR 4.38 [0.18, 105.82]) (Figure 4b).

#### 4.1.2 Inguinal Pain at Six Months or Longer After the Initial Hernia Surgery (Chronic Postoperative Pain)

**FIGURE 5:**
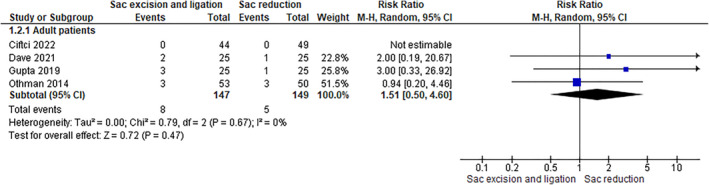
Chronic postoperative pain in cases of sac excision and ligation versus sac reduction.

In four randomized controlled trials (RCTs) involving 296 adult patients, a decreased pain rate of 3.35% was seen in the sac reduction group compared to the sac excision group (5.44%). However, this difference was not statistically significant (RR 1.51 [0.50, 4.60]) (Figure 5).

#### 4.1.3 PostOperative Pain (VAS Score) at 24 h

**FIGURE 6:**
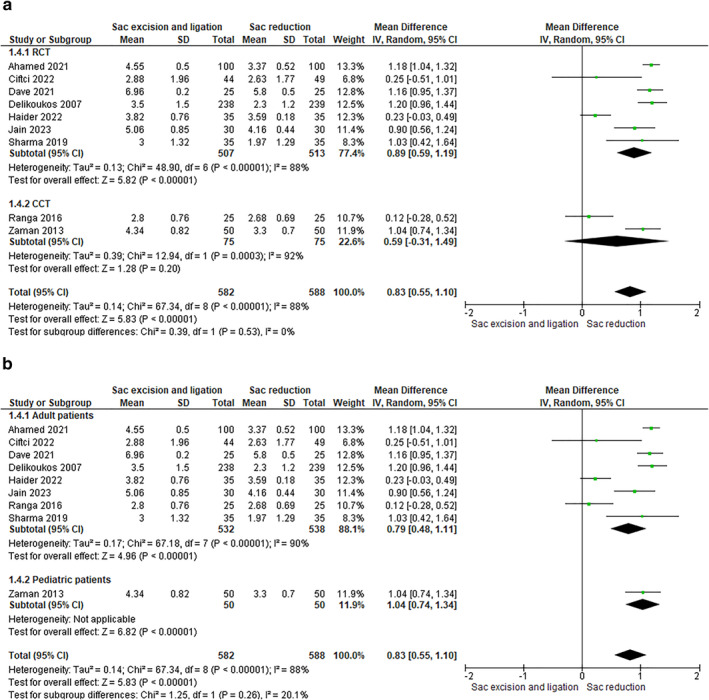
(a) Postoperative pain at 24 h after sac excision and ligation versus sac reduction. (b) Postoperative pain at 24 days after sac excision and ligation versus sac reduction.

A statistically significant lower median VAS pain at 24 h, evaluated in 9 studies (1170 patients), was observed in the sac reduction group compared to the sac excision group (RCTs: RR 0.89 [0.59, 1.19]–CCTs: RR 0.59 [‐0.31, 1.49]) (Figure 6a).

The trend of results was the same in analysis based on age (adults: RR 0.79 [0.48, 1.11]–pediatrics: RR 1.04 [0.74, 1.34]) (Figure 6b).

#### 4.1.4 PostOperative Pain (VAS Score) at 7 days

**FIGURE 7:**
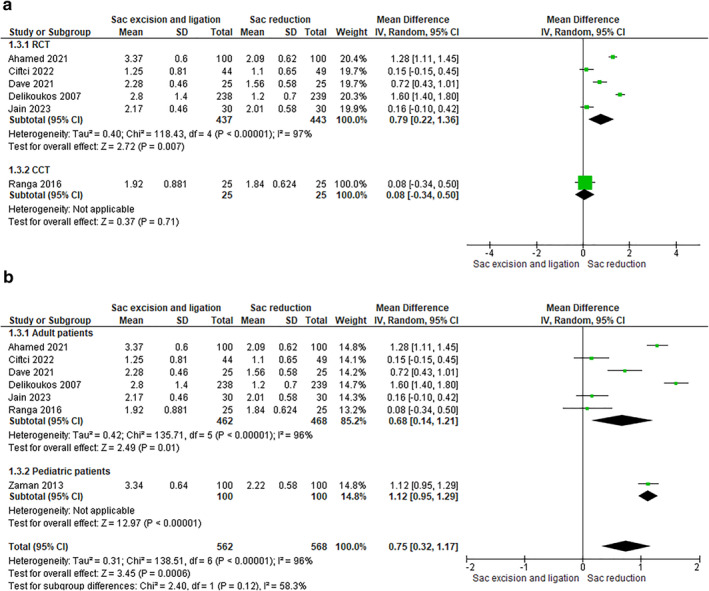
(a) Postoperative pain at 7 days after sac excision and ligation versus sac reduction. (b) Postoperative pain at 7 days after sac excision and ligation versus sac reduction.

Six RCTs, including 880 patients, and one CCT, including 50 patients, have evaluated this outcome. The sac reduction group showed a statistically significant lower median visual anal sphincter (VAS) pain at 7 days compared to the sac excision group (RCTs: RR 0.79 [0.22, 1.36]–CCTs: RR 0.08 [‐0.34, 0.50]) (Figure 7a).

The trend of results was same when performed an analysis based on age (9 studies, 1170 patients): adults: RR 0.68 [0.14, 1.21]–pediatrics: RR 1.12 [0.95, 1.29] (Figure 7b).

### 4.2 Secondary Outcomes

#### 4.2.1 Hematoma

**FIGURE 8:**
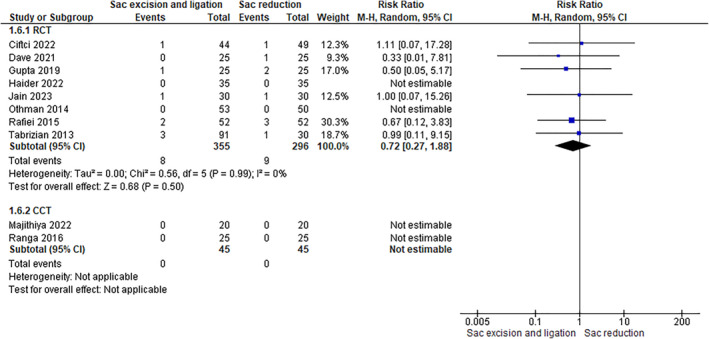
Hematoma after sac excision and ligation versus sac reduction.

This outcome was assessed in 10 studies (8 RCTs involving 651 patients and 2 CCTs involving 90 patients). The rate of this complication was the two groups (RCTs 2.55% in sac ligation vs. 3.04% in sac reduction), and data analysis revealed no significant difference (RCTs: RR 0.72 [0.27, 1.88]–CCTs: not estimable) (Figure 8).

#### 4.2.2 Wound Infection

**FIGURE 9:**
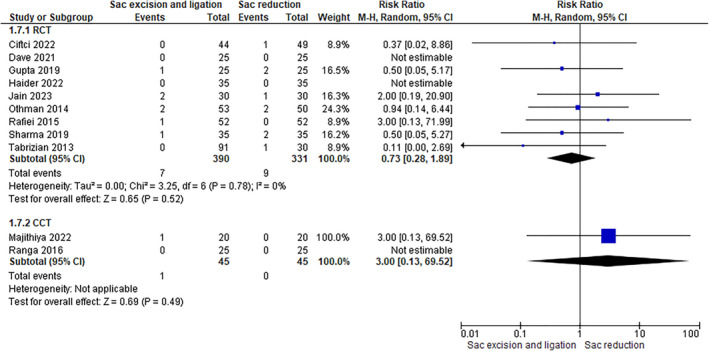
Wound infection after sac excision and ligation versus sac reduction.

The outcome has been assessed in 11 studies (9 RCTs involving 721 patients and 2 CCTs involving 90 patients). The rate of this complication was the same in the two groups (1.79% in excision and ligation and 2.72% in sac reduction), and the analysis did not reveal any significant differences (RCTs: RR 0.73 [0.28, 1.89]–CCTs: 3.00 [0.13, 69.52]) (Figure 9).

#### 4.2.3 Wound Seroma

**FIGURE 10:**
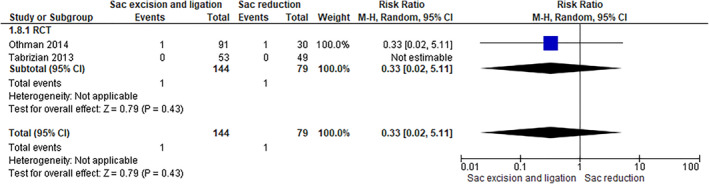
Wound seroma after sac excision and ligation versus sac reduction.

Two RCTs, including 223 patients, have assessed this outcome. Data analysis has shown a decrease of wound seroma in the group of patients who had ligature and excision (0.69%) compared to the group of patients who only underwent excision (1.27%). However, this result was not statistically significant (RR 0.33 [0.02, 5.11]; P 0.43) (Figure 10).

#### 4.2.4 Scrotal Edema

**FIGURE 11:**
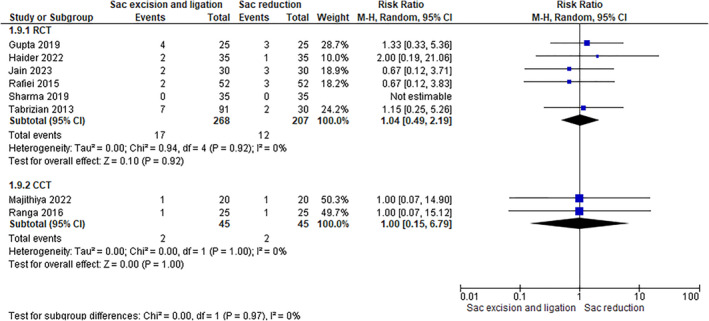
Scrotal edema after sac excision and ligation versus sac reduction.

Eight studies (6 RCTs, including 475 patients, and 2 CCTs including 90 patients were used to evaluate this outcome). Data analysis did not reveal any differences between the two groups: excision with high ligature (RCTs: 6.34%–CCTs: 4.44%) versus sac reduction (RCTs: 5.79–CCTs: 4.44%) (RCTs: RR 1.00 [0.15, 6.79]–CCTs: RR 1.00 [0.15, 6.79]) (Figure 11).

#### 4.2.5 Urinary Retention

**FIGURE 12:**
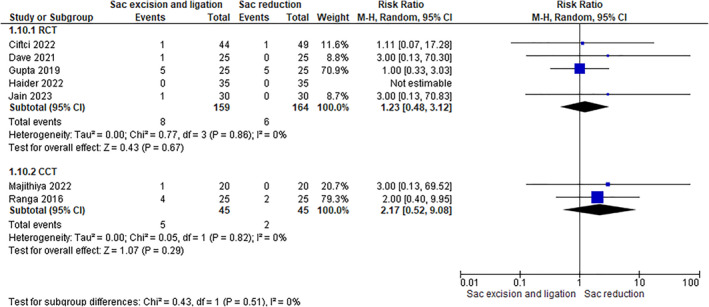
Urinary retection after sac excision and ligation versus sac reduction.

Eight studies (6 RCTs, including 475 patients, and 2 CCTs, including 90 patients) have assessed this outcome. The rate of this complication was the same between the two groups (6.07% in excision with ligature group vs. 5.55% in the sac reduction group), and data analysis did not reveal any differences (RCTs: RR 1.23 [0.48, 3.12]–CCTs: RR 2.17 [0.52, 9.08]) (Figure 12).

#### 4.2.6 Testicular Atrophy

This result has been assessed in 4 RCTs, involving 253 patients, and the analysis revealed no cases of testicular atrophy.

## 5 Discussion

Since the advent of Bassini's inguinal hernia surgery, most surgeons have deemed it essential to ligate the sac to avert hernia recurrence, despite the related increase in postoperative pain. Our extensive literature review has identified just two published randomized meta‐analyses of RCTs particularly addressing inguinal hernia repair: Kao et al. in 2015 [23] and Evans et al. in 2023 [24].

All studies conducted in the adult population focused solely on indirect hernias. The dimensions of the hernias were reported exclusively by Ciftci [9], who used the “European Hernia Society inguinal hernia classification” (EHS). Only Othman [16] also provided information on the dimensions of the internal ring. Othman used the Gilbert classification, which included 3 types: type 1 (normal internal ring), type 2 (internal ring ≯ 4 cm), and type 3 (internal ring ≥ 4 cm).

Within 14 research, surgeons only treated monolateral inguinal hernias, whereas only two studies documented the treatment of bilateral inguinal hernias. It is important to note that all surgical repairs of inguinal hernias were conducted as elective procedures.

All the papers, save for one [9], originated from the Middle East and Asia. This phenomenon may be attributed to the diminished efficacy of open tension‐free mesh surgery in the US and Europe, where minimally invasive techniques (laparoscopic and robotic) are being increasingly employed.

Concerning the documentation available for Kao [23], Evans [24], and our meta‐analyses, there were 12 randomized controlled trials (RCTs) and 3 controlled clinical trials (CCTs). The accessible data are considerably limited. The bulk of investigations on the adult population concentrated only on indirect, elective, unilateral mesh tension‐free inguinal hernia repair. Only two studies included bilateral inguinal hernia repair.

We observed a nonstatistically significant decrease in recurrence rates among adult and pediatric patients who underwent only sac reduction, compared to those who received sac excision along with ligation. A statistically significant reduction in median VAS pain was noted at 24 h and 7 days following surgery in the sac reduction cohort. No statistically significant difference has been seen between the two study groups for the incidence of postoperative wound seroma, wound hematoma, wound infection, scrotal edema, and urinary tract infection. No cases of testicular atrophy have been recorded.

A possible bias in the research conducted by Yu Kao [23] is that it analyzed merely five studies published between 1984 and 2014, which had patient samples ranging from 50 to 467 individuals. Moreover, one study [25] incorporated laparoscopic hernia repair, which, as previously mentioned, is a fundamentally distinct surgical approach, rendering its outcomes incomparable to those achieved by open surgery. While a unified opinion on this issue is lacking, laparoscopic inguinal repair appears to be correlated, particularly in pediatric patients, with reduced postoperative pain compared to open inguinal hernia repair [26]. This bias may have influenced the conclusions of Kao [23], who asserts that “hernia sac ligation was correlated with increased postoperative pain."

Evans' meta‐analysis [26] is more current and yet evaluates only six trials that exclusively involve adult patients. Kao's study [23], akin to our research, revealed no significant disparity in recurrences and postoperative complications between the two study cohorts. The sole distinction between the two meta‐analyses is that our study identified postoperative discomfort, especially 7 days post‐surgery, to be significantly less severe and less prevalent in cases involving sac invagination. Evans' findings closely corresponded with our research, revealing only slight discrepancies: In the context of sac ligation, early (6 h) postoperative pain assessed by VAS was markedly higher (*p* ≤ 0.00001) and persisted at elevated levels at 12 h (*p* = 0.001), 24 h (*p* ≤ 0.00001), and on the seventh postoperative day (*p* = 0.009).

To formulate definitive guidelines for the management of inguinal hernia sac in tension‐free mesh operations, more comprehensive meta‐analyses, encompassing a larger quantity of higher quality randomized controlled trials (RCTs) and controlled clinical trials (CCTs), are likely necessary. Conducting such a study may be problematic due to the decreasing incidence of open inguinal hernia repairs worldwide, attributed to the rise of minimally invasive surgical procedures.

## 6 Conclusions

This meta‐analysis of randomized controlled trials (RCTs) and controlled clinical trials (CCTs) indicates that, 1 year or more post‐hernia surgery, there is no statistically significant difference in recurrence rates between patients who underwent sac reduction and those who received sac excision and ligation. Unfortunately the follow‐up period reported in these trials was different, making more difficult to reach definitive conclusions on recurrence rate.

Subgroup analysis categorized by patient age produced analogous outcomes. The assessment of two therapies for the sac in pediatric patients indicated that primary and secondary results were statistically similar between the two groups. Nonetheless, ligation of the sac may result in surgical problems, whereas transection of the sac may spontaneously occlude in juvenile patients. This study tacitly endorses the excision of the sac without ligation as the “gold standard” treatment for pediatric patients.

This study's findings demonstrate that different surgical interventions for the inguinal hernia sac have comparable primary and secondary outcomes in both adults and children. The sole distinction is that individuals with sac invagination saw milder and less frequent early postoperative discomfort.

There is no prevailing agreement regarding the management of the inguinal hernia sac (reduction vs. resection) in adults. However this meta‐analysis further supports our clinical preference for lowering the sac without incision during elective open inguinal hernia surgery using a tension‐free mesh in adult patients, aiming to minimize postoperative discomfort incidence and severity.

## Author Contributions

**Roberto Cirocchi:** conceptualization, data curation, formal analysis, project administration, resources, software, supervision, validation, visualization, writing–original draft. **Georgi I. Popivanov:** formal analysis, investigation, methodology. **Maria Chiara Cianci:** writing–review & editing. **Antonino Morabito:** writing–review & editing. **Matteo Matteucci:** formal analysis, investigation, methodology, project administration, resources, software, supervision, validation, visualization, writing–original draft. **Sara Lauricella:** writing–review & editing. **Diletta Cassini:** writing–review & editing. **Carlo Boselli:** writing–review & editing. **Ivan Szergyuk:** writing–review & editing. **Giovanni Domenico Tebala:** writing–review & editing. **Antonia Rizzuto:** writing–review & editing. **Paolo Bruzzone:** conceptualization, data curation, formal analysis, investigation, methodology, project administration, resources, software, supervision, validation, visualization, writing–original draft.

## Ethics Statement

The authors have nothing to report.

## Consent

The authors have nothing to report.

## Conflicts of Interest

The authors declare no conflicts of interest.

## Human Subjects/Animal Subjects

Not relevant.

## Data Availability

The data used to support the finding of this study are included within the article. Further inquiries can be directed to the corresponding author.
